# HDncRNA: a comprehensive database of non-coding RNAs associated with heart diseases

**DOI:** 10.1093/database/bay067

**Published:** 2018-07-24

**Authors:** Wen-Jing Wang, Yu-Mei Wang, Yi Hu, Qin Lin, Rou Chen, Huan Liu, Wen-Ze Cao, Hui-Fang Zhu, Chang Tong, Li Li, Lu-Ying Peng

**Affiliations:** 1 Key Laboratory of Arrhythmias, Ministry of Education, Tongji University School of Medicine, No. 150, Jimo Road, Pudong New District, Shanghai, China; 2 Research Center for Translational Medicine, Shanghai East Hospital, Tongji University School of Medicine, No. 150, Jimo Road, Pudong New District, Shanghai, China; 3 Department of Pathology and Pathophysiology, Tongji University School of Medicine, No. 1239, Siping Road, Yangpu District, Shanghai, China

## Abstract

Heart diseases (HDs) represent a common group of diseases that involve the heart, a number of which are characterized by high morbidity and lethality. Recently, increasing evidence demonstrates diverse non-coding RNAs (ncRNAs) play critical roles in HDs. However, currently there lacks a systematic investigation of the association between HDs and ncRNAs. Here, we developed a Heart Disease-related Non-coding RNAs Database (HDncRNA), to curate the HDs-ncRNA associations from 3 different sources including 1904 published articles, 3 existing databases [the Human microRNA Disease Database (HMDD), miR2disease and lncRNAdisease] and 5 RNA-seq datasets. The HDs-ncRNA associations with experimental validations curated from these articles, HMDD, miR2disease and part of data from lncRNAdisease were ‘direct evidence’. Relationships got from high-through data in lncRNAdisease and annotated differential expressed lncRNAs from RNA-seq data were defined as ‘high-throughput associations’. Novel lncRNAs identified from RNA-seq data in HDs had least credibility and were defined as ‘predicted associations’. Currently, the database contains 2304 HDs-ncRNA associations for 133 HDs in 6 species including human, mouse, rat, pig, calf and dog. The database also has the following features: (i) A user-friendly web interface for browsing and searching the data; (ii) a visualization tool to plot miRNA and lncRNA locations in the human and mouse genomes; (iii) information about neighboring genes of lncRNAs and (iv) links to some mainstream databases including miRbase, Ensemble and Fantom Cat for the annotated lncRNAs and miRNAs. In summary, HDncRNA provides an excellent platform for exploring HDs related ncRNAs.

Database URL: http://hdncrna.cardiacdev.com

## Introduction

Heart disease (HD) is a leading cause of worldwide morbidity and mortality (1) and may attribut to dysfunction of gene expression at different levels such as epigenetic, transcriptional and translational regulations (2). One primary goal in HD research is to clarify molecular mechanisms.

Non-coding RNAs (ncRNAs) is a series of RNAs that generally do not encode proteins. By length or other characteristics, ncRNAs are classified into several categories including microRNA (miRNA), long ncRNAs (lncRNA), circular RNA (circRNA), small interfering RNA (siRNA), small nucleolar RNAs (snoRNA) etc (3). Recent studies indicated that over 70% of human genome could be transcribed into ncRNAs (4) and a number of the molecules have been found to be involved in the cardiac diseases through affecting cardiac-related gene expression and could be the potential targets of cardiac disease (5). For example, miR-133 has been showed to mediate the progress of cardiac hypertrophy by directly regulating RhoA and Cdc42 (6). miR-1 was down-regulated in patients with symptomatic heart failure by regulating pacemaker channel genes HCN2 and HCN4 (7). Dysregulated miR-21 in cardiac fibroblast contributes to myocardial infarction by stimulating MAP kinase signaling in fibroblasts (8). lncRNA *Chrf* acts as endogenous ‘sponge’ of miR-489 to block pro-hypertrophic miR-489 (9). In early stage of cardiac hypertrophy, lncRNA *Chaer* could function via directly interacting with PRC2 and inhibiting histone H3 lysine 27 methylation at the promoter regions of genes involved in the disease (10). A number of ncRNAs in HDs were discovered in various studies (11, 12). Even though many ncRNAs that implicate in heart disorders have been analyzed, collection platform involved is still lack.

As the development of computer science, ncRNA databases have been easily used by researchers as powerful tools by their advantages: logical relationship between records and data, low redundancy and convenience of management. However, databases revel relationships between heat diseases and ncRNAs are limited. With the research progress of ncRNAs, several comprehensive databases about ncRNAs such as miRBase (13), lncRNAdb (14), GENCODE (15) and NONCODE (16) have been well developed. Some disease-related ncRNA databases such as the Human microRNA Disease Database (HMDD) (17), miR2disease (18) and lncRNAdisease (19) have emerged as well. Moreover, the recent progresses in RNA-seq technologies make it possible to predict ncRNA associated with HDs.

Therefore, a systematic curation of the HDs-ncRNA associations from these sources will facilitate further research in HDs. In this study, we constructed a database Heart Disease-related Non-coding RNAs Database (HDncRNA) by assembling and characterizing the associations between ncRNAs and HDs derived from the aforementioned sources including literatures, the ncRNA databases (HMDD, miR2disease and lncRNAdisease) and high-throughput ncRNA datasets in Gene Expression Omnibus (GEO) (20). HDs-ncRNA associations with experiment validation curated from published articles and above three ncRNA databases were defined as ‘direct evidence’, ‘high-throughput associations’ were got from high-through data in lncRNAdisease and annotated differential expressed lncRNAs from GEO-sourced RNA-seq datasets, and associations predicted from high-throughput RNA-seq data were defined ‘predicted correlations’.

## Materials and methods

**Figure 1.**
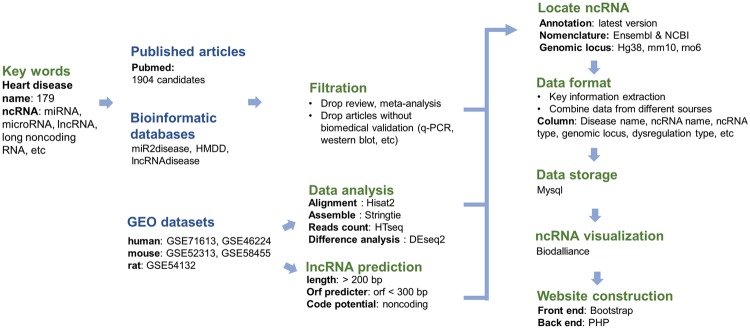
The whole pipeline of database construction. The titles in blue mark the sources of data in HDncRNA, and the titles in green represent steps of database construction.

To ensure the integrity and reliability of the data, three sources data, publications, existing ncRNAdatabases and GEO datasets were collected. Besides, we made reliability ratings for all sources of data. We defined ‘direct evidence’ were items which methods include experimental validation like principal component regression (PCR), western blot, luciferase assay, etc. ‘high-throughput associations’ were items which methods include annotated ncRNAs from microarray analysis, high-throughput sequencing and genome-wide association study (GWAS). Items include predicted novel ncRNAs were classified as ‘predicted associations’. Among three sources of data, ‘direct evidence’ was extracted from published articles, miR2disease, HMDD and part of the content in lncRNAdisease. ‘High-throughput associations’ were got from lncRNAdisease and RNA-seq datasets in GEO. Items include predicted novel lncRNAs got from GEO was defined as ‘predicted associations’. The whole pipeline for database construction from three sources can be seen in Figure 1.

### Data collection from articles in PubMed

Firstly, we collected HD keywords from 10th edition of International Classification of Diseases (ICD-10) (21) and Disease Ontology (22). A total of 179 appellations of HDs were obtained during this process. As for ncRNA key words, we searched with following names: ‘microRNA’, ‘miRNA’, ‘long non-coding RNA’, ‘lncRNA’, ‘circular RNA’ and ‘circRNA’. By combining 179 disease names and these 6 ncRNA keywords, we fetched articles in PubMed which contain these combined key words in Text Word area. The search is ended on 31January 2018. During both combination and search progress, Perl scripts were frequently used.

Then, irrelevant articles were filtered by reading manually. We filtered articles by following criterions: firstly, reviews and meta-analysis were removed by manual review; secondly, by reading abstract or entire text, articles whose methods do not contain biomedical experimental validation (like q-PCR, western blot, etc.) were dropped as well. Thus, we kept articles related to HDs and ncRNAs and extracted key information as ‘direct evidence’.

### Data collection from ncRNA databases

With the same key words used in PubMed, we then searched items in miR2disease, HMDD and lncRNAdisease. Afterwards, we deleted items which come from reviews, meta-analysis or retracted articles. For miR2disease and HMDD, we only kept articles with ‘direct evidence’. Differently, we retained both ‘direct evidence’ and ‘high-throughput associations’ from lncRNAdisease in order to provide more abundant information about HDs–lncRNA relations.

### Data analysis and prediction from RNA-seq datasets

Five RNA-seq datasets were downloaded from GEO: GSE71613, GSE46224, GSE52313, GSE58455 and GSE54132. The RNAs are all taken from heart tissue in the five datasets, for the specificity of tissue ensures the reliability of further analysis. Details about these five datasets were shown in Table 1.

**Table 1.** The five RNA-seq datasets used in HDncRNA

Series	Species	Samples	Tissue	Conditions	Condition details (numbers)
GSE71613	Human	8	Heart	3	Control (4), restrictive cardiomyopathy (2), dilated cardiomyopathy (2)
GSE46224	Human	8	Heart	2	Non-falling control (4), ICM (4)
GSE52313	Mouse	2	Heart	2	Sham (4), myocardial infarction (4)
GSE58455	Mouse	8	Heart	8	Sham (4), TAC (4)
GSE54132	Rat	6	Heart	2	Control (3), myocardial infarction (2)

ICM: Ischemic cardiomyopathy; TAC: Transverse aortic constriction.

**Figure 2.**
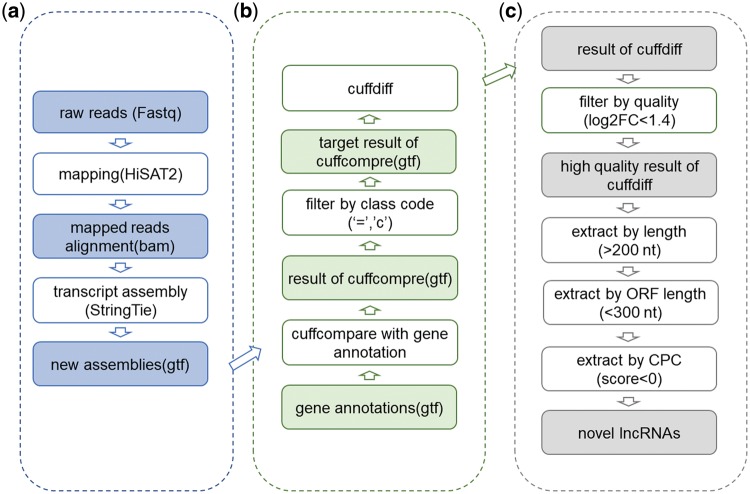
The workflow of lncRNA analysis and prediction. (**a**) Initial assembly. Raw reads were mapped to reference genome of corresponding species. When Cufflinks ran, −*g* parameter was dropped to find new transcripts. (**b**) Cufflinks toolkit usage for novel lncRNAs detection. During novel lncRNA prediction, Cuffcompare and Cuffdiff were used. (**c**) Predict potential novel lncRNAs from results got from Cufflinks. During the prediction process, threshold of log2FC of FPKM was set as 1.4, length threshold was set as 200 nt, threshold of ORF length by ORF predictor (29) was set as 300 nt and threshold score of CPC (coding potential calculator) (30) was set as 0.

We reannotated long non-coding RNAs (lncRNAs) and predicted novel lncRNAs. Firstly, reads from each sample were assessed for quality by FastQC (23) and mapped to the latest reference genomes (hg38, mm10, rno6) depending on different species by HISAT2 (24). Further, mapped reads alignments in bam format were got. Secondly, StringTie (25) was used to assemble alignments into full and partial transcripts and created multiple isoforms. After that progress, new assemblies were got and compared with gene annotations in gtf format. With Cuffdiff (26), we extracted significantly differentially expressed genes and annotated them to get known protein coding genes and lncRNAs. All newly annotated lncRNAs were contained in ‘predicted associations’. Then following previous methods (27), we predicted novel differentially expressed lncRNAs after filtering based on the length of sequence, coding potential and open reading frame (ORF) length. Detailed lncRNA prediction methods can be seen in Figure 2. Perl scripts applied to filter lncRNA have been uploaded to Github (https://github.com/TJWenjing/HDncRNA). Since these five datasets were not processed with Rnase treatment and the lack of annotation files (28), we did not further predict circRNA with these data.

### Database and website construction

To extract key information from three sources data mentioned above, gene symbol, gene locus and latest id of miRNAs were got from miRbase, and same information of lncRNAs were got from Ensembl (31). However, gene locus of circRNAs was not provided temporarily. Other information like species, tissue, methods of experiment, summary of HDs–ncRNA relationship and direct target genes were got from articles and databases. As studies showed that lncRNAs can act as *cis*-element, we also found neighbor genes of all lncRNAs and made a table. By referring previous articles (32, 33), we defined genes only locate within 10 kb upstream or downstream of target lncRNAs as neighbor genes.

All data sources were integrated into database by MySQL, and the website was built by HTML, Javascript and PHP. Visualization model was added for some ncRNAs based on Biodalliance (34) and neighbor genes information was connected to lncRNAs as well.

## Results

### Database content

By 31 January 2018, we got 1904 potential articles from PubMed using key words searching. After filtering and manual annotation detailed in the Materials and methods, we derived 438 articles from which we identified 852 ncRNA associations with 123 HDs. We also collected 632 experiment-supported associations from miR2disease, HMDD and lncRNAdisease. From the RNA-seq data in GEO, we identified 820 lncRNA-HD associations which included 356 predicted novel ones (Supplementary Table S1). It is noteworthy that 29 publication-based associations overlap database-based ones, which proved the credibility of data. We finally removed redundant data.

Currently, the database includes 2304 HD-ncRNA associations in 6 species with 13 from calf, dog and pig and the rest from human, mouse and rat. Every item contains very detailed information including disease name, ncRNA name, species, ncRNA expression (e.g. up-regulated, down-regulated), experimental methods (e.g. microarray or Real-time PCR), experimentally validated targets of ncRNAs, detected tissue (e.g. heart, blood), details about HD–ncRNA relationships and associated literature (PubMed ID, title and publication year). For lncRNAs, information about their neighboring genes is also provided. Table 2 shows the statistics of all data sources utilized for creating the database.

**Table 2.** The entries in the HDncRNA database

Data source	miRNA				lncRNA				circRNA			Total
	Human	Mouse	Rat	Other	Human	Mouse	Rat	Other	Human	Mouse	Rat	
Article	438	162	128	11	53	35	15	2	3	4	1	852
Database	151	122	45	4	21	288	1	0	0	0	0	632
GEO	0	0	0	0	268	520	32	0	0	0	0	820
Total	1061				1235				8			2304

Among the 2304 items, 820 were identified from RNA-seq datasets and 356 involve predicted novel lncRNAs. Notably, H19 was identified in both GSE46224 and three articles. Table 3 shows detailed distribution of those novel lncRNAs.

**Table 3.** Number of annotated and - novel lncRNAs associated with HDs in RNA-seq datasets

	Human	Mouse	Rat
Annotated lncRNA	226	228	10
Unannotated lncRNA	42	292	22
Total	268	520	32

### Heart diseases covered by HDncRNA

**Figure 3.**
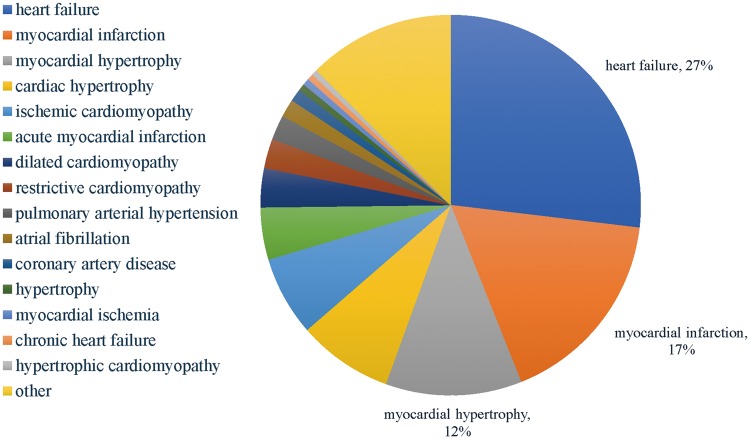
The distribution of the HDs in the HDncRNA.

Figure 3 shows the distribution of the HDs in the database. The top 5 HDs in over 60% associations in the database are heart failure, myocardial infarction, myocardial hypertrophy, cardiac hypertrophy and ischemic cardiomyopathy, indicating the existing studies of HDs and ncRNAs are more focused on heart failure, myocardial infarction and cardiac hypertrophy.

### User interface

**Figure 4.**
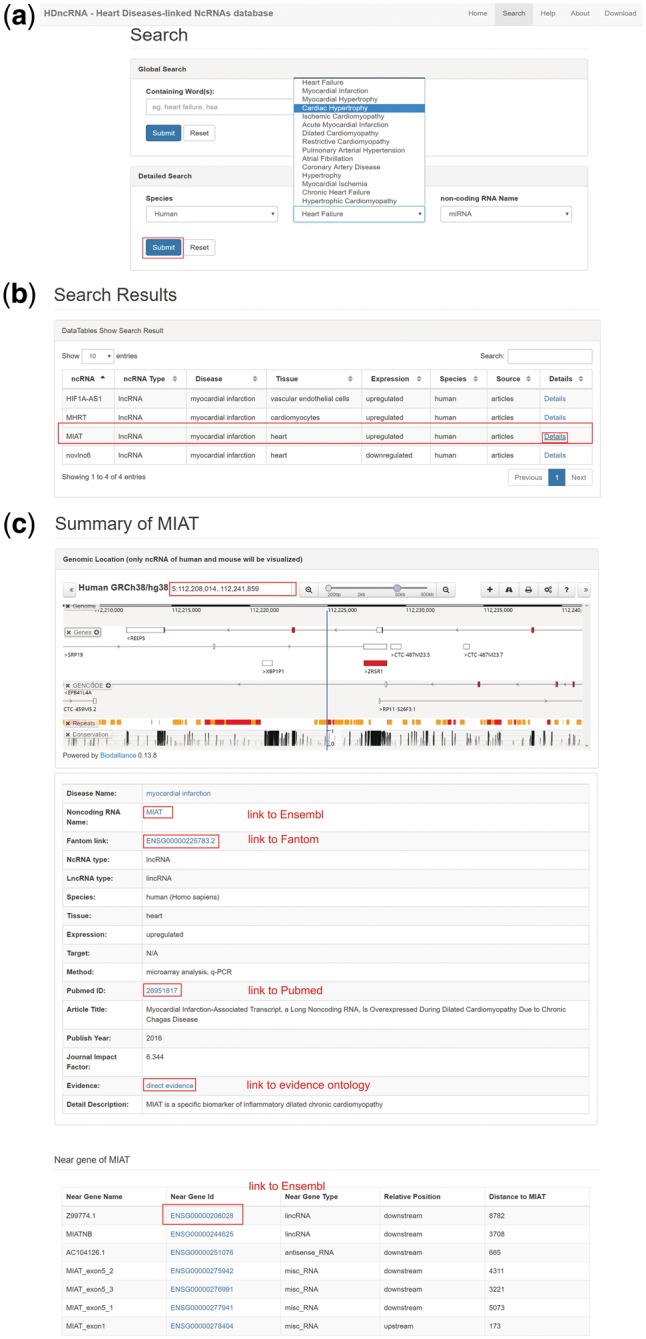
The workflow of HDncRNA. (**a**) Search page of HDncRNA, in which two search methods are provided. (**b**) Table contains search results. In the table, data can be ordered by every column, search in table is also supported. (**c**) Details webpage of the lncRNA MIAT. The top model is visualization of the MIAT, the genome locus and nearby biological molecules can be seen. In the middle model, detailed information of MIAT is provided. Neighbor genes of MIAT are shown in the bottom model. Relative position and distance between each two genes are provided as well.

HDncRNA provides a user-friendly website to query the data. The home page gives a brief introduction about HD and ncRNAs while querying the database is conducted on the search page (Figure 4a). Two search methods, global search and detailed search, were provided. In global search, users can input target information by texting any keywords (e.g. species/disease name/ ncRNA type/ncRNA name) in the search bar. In detailed search, three conditions are provided for users to get precise information (Figure 4a).

The search results of HDncRNA are shown in Figure 4b. These include a table of search results. A Search bar top right the webpage allows users to get target information effectively. The table includes basic information such as disease names, ncRNAs names and PubMed ID as well as more valuable details about expression of ncRNAs in disease, gene locus of ncRNAs, target biological factors of ncRNAs and conclusion about relations between ncRNAs and HDs. In addition, the source of each association is provided as well.

When users click the button ‘details’, detailed information about ncRNAs and diseases will be shown in a new page. Figure 4c shows an example of myocardial infarction associated transcript (MIAT) related myocardial infarction. The genome locus of MIAT is visualized to facilitate exploration of its neighboring genes. The table at bottom shows the nearby genes of MIAT (10 kb upstream or downstream). By simple clicking the ensemble id of these genes, users will be redirected to Ensemble. In addition, for annotated lncRNAs in human, we added hyperlinks to Fantom Cat (35), which provide affluent features about human lncRNAs. For diseases included in Disease Ontology (22), we linked them to target webpages of Disease Ontology. In addition, description of evidence information is linked to Evidence Ontology (36), where users can better understand evidence we used.

HDncRNA also allows all data shown in the website can be downloaded easily in ‘Download’ page. As ncRNAs are still a hotspot in life sciences and medical area, ‘Help’ page gives a detailed introduction as well as tutorial for users.

## Discussion

One advantage of our database is unification of different kinds of data. In fact, we combined data from published articles, database and RNA-seq datasets in GEO. Firstly, mining valuable information from the literature can be challenging because of the dispersed information. Besides, although existing databases such as miR2Disease, HMDD and lncRNADisaese all focus on relationships between diseases and ncRNAs, the data sources are not very clearly classified. More importantly, the credibility of the data in these databases is not provided. In this study, we built up a database with abundant and relative high credible data by we unifying the three sources. To meet the different needs of users, we then classified each item according to evidence it contained.

As epigenetics and bioinformatics, nomenclature and locus of some ncRNAs all evolves rather quickly, it is essential to update the information periodically. Taking Ensemble as an example, there are 7340 lincRNAs in GRCh37.75 and 7493 lincRNAs in GRCh38.90. Some ncRNA databases hold computationally predicted associations, but others only include manually curated experimentally validated data sets. For example, miR2Disease only includes publication-based information by 2009. Although lncRNADisease lacks rating system and detailed introduction about lncRNAs, it provides both experiment-verified and predicted disease-related lncRNAs. Thus, it is meaningful to integrate data with consistent and latest annotation. HDncRNA was constructed with latest annotation comes from miRbase (13), National center for biotechnology information (NCBI) (37), Noncode (16) and Ensemble (31), ensuring the consistency and high quality of annotation for all ncRNAs. By using the latest annotation, we can provide more comprehensive information in HDncRNA.

We also compared HDncRNA with another database focusing on links between ncRNAs and cardiovascular diseases, Cardio_ncRNA (38). HDncRNA is more focused on experimentally validated data and has high reliability. In addition, more lncRNAs and related information are provided in HDncRNA. Apart from this, by adding data analyzed from RNA-seq data, copious differential expressed lncRNAs found between healthy and sick hearts are included, and some of them are novel lncRNAs produced by our prediction. As lncRNAs act as *cis*-acting element, we also add neighbor genes of them. Another characteristic of HDncRNA is the convenient visualization model for locating lncRNA and check its neighbor genes on the genome.

Interestingly, we found a known lncRNA, H19, appeared in 10 items among 3 sources data. H19 not only appears in six published articles, but also shows in three items of database-sourced data and one result of RNA-seq data. This presents the consistency of different sources data and sufficient evidence which shows strong relationship between H19 and HDs.

## Conclusion

In this study, we developed a database, HDncRNA, to provide comprehensive and precise data about the associations between HDs and ncRNAs from a variety of sources. Reference papers were provided to perform specific ncRNAs in HDs. In the future, we will update the database annually. We will also enrich the database with more effective prediction tools. HDncRNA will play an important role in studying ncRNA related mechanism in HDs.

## Supplementary data

Supplementary data are available at *Database* Online.

## Supplementary Material

Supplementary Data
