# Supplementary material for: HDncRNA: a comprehensive database of non-coding RNAs associated with heart diseases
Source: Database (Oxford). 2018 Jul 24;2018:bay067. doi: 10.1093/database/bay067 (PMC6059085; doi:10.1093/database/bay067)
Supplement: Supplementary Data [file bay067_table_s1.docx]

**Table S1 Novel LncRNA-HD associations predicted from RNA-seq datasets**

| **ncRNA_type** | **chr** | **start** | **end** | **species** | **change_in_disease** | **tissue** | **p_value** | **source** | **grade** |  |
| --- | --- | --- | --- | --- | --- | --- | --- | --- | --- | --- |
| lncRNA | 1 | 72886780 | 72887134 | rat | downregulated | heart | 0.00005 | GSE54132 | predicted associations | |
| lncRNA | 1 | 140898135 | 140898560 | rat | upregulated | heart | 0.00005 | GSE54132 | predicted associations | |
| lncRNA | 3 | 138710593 | 138710877 | rat | downregulated | heart | 0.0004 | GSE54132 | predicted associations | |
| lncRNA | 4 | 117542468 | 117543401 | rat | downregulated | heart | 0.00005 | GSE54132 | predicted associations | |
| lncRNA | 4 | 180846117 | 180846557 | rat | downregulated | heart | 0.00005 | GSE54132 | predicted associations | |
| lncRNA | 4 | 180846117 | 180846557 | rat | downregulated | heart | 0.0002 | GSE54132 | predicted associations | |
| lncRNA | 5 | 156751981 | 156752262 | rat | downregulated | heart | 0.00005 | GSE54132 | predicted associations | |
| lncRNA | 6 | 8165729 | 8166039 | rat | upregulated | heart | 0.00025 | GSE54132 | predicted associations | |
| lncRNA | 6 | 91475224 | 91476608 | rat | upregulated | heart | 0.00005 | GSE54132 | predicted associations | |
| lncRNA | 7 | 287397 | 287756 | rat | downregulated | heart | 0.00005 | GSE54132 | predicted associations | |
| lncRNA | 7 | 733670 | 734454 | rat | downregulated | heart | 0.00005 | GSE54132 | predicted associations | |
| lncRNA | 7 | 1678262 | 1678536 | rat | downregulated | heart | 0.00005 | GSE54132 | predicted associations | |
| lncRNA | 7 | 22558228 | 22558458 | rat | upregulated | heart | 0.0004 | GSE54132 | predicted associations | |
| lncRNA | 7 | 58617678 | 58618014 | rat | downregulated | heart | 0.0002 | GSE54132 | predicted associations | |
| lncRNA | 7 | 99481338 | 99481600 | rat | downregulated | heart | 0.00005 | GSE54132 | predicted associations | |
| lncRNA | 7 | 109270677 | 109271105 | rat | downregulated | heart | 0.00025 | GSE54132 | predicted associations | |
| lncRNA | 9 | 43551231 | 43551532 | rat | downregulated | heart | 0.00025 | GSE54132 | predicted associations | |
| lncRNA | 10 | 85864602 | 85865262 | rat | upregulated | heart | 0.00005 | GSE54132 | predicted associations | |
| lncRNA | 10 | 109071359 | 109071583 | rat | downregulated | heart | 0.00005 | GSE54132 | predicted associations | |
| lncRNA | 10 | 109071359 | 109071583 | rat | downregulated | heart | 0.0001 | GSE54132 | predicted associations | |
| lncRNA | 14 | 34019887 | 34020736 | rat | downregulated | heart | 0.00005 | GSE54132 | predicted associations | |
| lncRNA | 14 | 34019887 | 34020736 | rat | downregulated | heart | 0.00015 | GSE54132 | predicted associations | |
| lncRNA | 1 | 9537867 | 9621245 | mouse | downregulated | heart | 0.00005 | GSE52313 | predicted associations | |
| lncRNA | 1 | 12682562 | 12851436 | mouse | upregulated | heart | 0.00005 | GSE52313 | predicted associations | |
| lncRNA | 1 | 21230687 | 21255495 | mouse | downregulated | heart | 0.0002 | GSE52313 | predicted associations | |
| lncRNA | 1 | 36364615 | 36385869 | mouse | upregulated | heart | 0.00015 | GSE52313 | predicted associations | |
| lncRNA | 1 | 43203630 | 43204053 | mouse | downregulated | heart | 0.0049 | GSE58455 | predicted associations | |
| lncRNA | 1 | 45431108 | 45560156 | mouse | upregulated | heart | 0.00005 | GSE52313 | predicted associations | |
| lncRNA | 1 | 51465167 | 51465820 | mouse | upregulated | heart | 0.0007 | GSE58455 | predicted associations | |
| lncRNA | 1 | 60867912 | 60972663 | mouse | downregulated | heart | 0.0002 | GSE52313 | predicted associations | |
| lncRNA | 1 | 69108086 | 69108463 | mouse | downregulated | heart | 0.00415 | GSE58455 | predicted associations | |
| lncRNA | 1 | 71288854 | 71354069 | mouse | downregulated | heart | 0.0001 | GSE52313 | predicted associations | |
| lncRNA | 1 | 71632093 | 71699837 | mouse | upregulated | heart | 0.00005 | GSE52313 | predicted associations | |
| lncRNA | 1 | 75211549 | 75215845 | mouse | downregulated | heart | 0.0001 | GSE52313 | predicted associations | |
| lncRNA | 1 | 78813353 | 78816601 | mouse | upregulated | heart | 0.0005 | GSE52313 | predicted associations | |
| lncRNA | 1 | 92390717 | 92391032 | mouse | downregulated | heart | 0.0025 | GSE58455 | predicted associations | |
| lncRNA | 1 | 121312052 | 121317547 | mouse | upregulated | heart | 0.0001 | GSE52313 | predicted associations | |
| lncRNA | 1 | 135806172 | 135818003 | mouse | upregulated | heart | 0.00005 | GSE52313 | predicted associations | |
| lncRNA | 1 | 136364885 | 136365104 | mouse | upregulated | heart | 0.00005 | GSE58455 | predicted associations | |
| lncRNA | 1 | 139959869 | 140071865 | mouse | upregulated | heart | 0.00005 | GSE52313 | predicted associations | |
| lncRNA | 1 | 144277848 | 144278365 | mouse | downregulated | heart | 0.0002 | GSE58455 | predicted associations | |
| lncRNA | 1 | 151676757 | 151808419 | mouse | upregulated | heart | 0.00005 | GSE52313 | predicted associations | |
| lncRNA | 1 | 172574560 | 172612904 | mouse | upregulated | heart | 0.00005 | GSE52313 | predicted associations | |
| lncRNA | 1 | 180459102 | 180869278 | mouse | upregulated | heart | 0.0009 | GSE52313 | predicted associations | |
| lncRNA | 1 | 182020043 | 182020273 | mouse | upregulated | heart | 0.00465 | GSE58455 | predicted associations | |
| lncRNA | 1 | 191941696 | 192021975 | mouse | downregulated | heart | 0.00065 | GSE52313 | predicted associations | |
| lncRNA | 2 | 10075247 | 10178143 | mouse | upregulated | heart | 0.00005 | GSE52313 | predicted associations | |
| lncRNA | 2 | 14988168 | 14988612 | mouse | downregulated | heart | 0.0017 | GSE58455 | predicted associations | |
| lncRNA | 2 | 22744729 | 22744989 | mouse | downregulated | heart | 0.00005 | GSE58455 | predicted associations | |
| lncRNA | 2 | 22744729 | 22744989 | mouse | downregulated | heart | 0.00005 | GSE58455 | predicted associations | |
| lncRNA | 2 | 22744729 | 22744989 | mouse | downregulated | heart | 0.00015 | GSE58455 | predicted associations | |
| lncRNA | 2 | 22744729 | 22744989 | mouse | downregulated | heart | 0.00015 | GSE58455 | predicted associations | |
| lncRNA | 2 | 26934879 | 26964498 | mouse | upregulated | heart | 0.00005 | GSE52313 | predicted associations | |
| lncRNA | 2 | 27741623 | 27895024 | mouse | upregulated | heart | 0.00005 | GSE52313 | predicted associations | |
| lncRNA | 2 | 32269529 | 32306679 | mouse | upregulated | heart | 0.0002 | GSE52313 | predicted associations | |
| lncRNA | 2 | 55288148 | 55450551 | mouse | downregulated | heart | 0.00075 | GSE52313 | predicted associations | |
| lncRNA | 2 | 57984727 | 58012505 | mouse | upregulated | heart | 0.00005 | GSE52313 | predicted associations | |
| lncRNA | 2 | 68699558 | 68952316 | mouse | upregulated | heart | 0.00005 | GSE52313 | predicted associations | |
| lncRNA | 2 | 75639246 | 75640028 | mouse | upregulated | heart | 0.00025 | GSE58455 | predicted associations | |
| lncRNA | 2 | 79674609 | 79879044 | mouse | upregulated | heart | 0.00005 | GSE52313 | predicted associations | |
| lncRNA | 2 | 84976522 | 84980078 | mouse | downregulated | heart | 0.0002 | GSE52313 | predicted associations | |
| lncRNA | 2 | 86018878 | 86019199 | mouse | upregulated | heart | 0.00005 | GSE58455 | predicted associations | |
| lncRNA | 2 | 102390241 | 102483199 | mouse | upregulated | heart | 0.00005 | GSE52313 | predicted associations | |
| lncRNA | 2 | 113671723 | 113688824 | mouse | upregulated | heart | 0.0008 | GSE52313 | predicted associations | |
| lncRNA | 2 | 129093964 | 129122904 | mouse | upregulated | heart | 0.00005 | GSE52313 | predicted associations | |
| lncRNA | 2 | 163646585 | 163658912 | mouse | upregulated | heart | 0.00005 | GSE52313 | predicted associations | |
| lncRNA | 2 | 167028225 | 167066403 | mouse | upregulated | heart | 0.00005 | GSE52313 | predicted associations | |
| lncRNA | 2 | 177511161 | 177512357 | mouse | downregulated | heart | 0.0048 | GSE58455 | predicted associations | |
| lncRNA | 2 | 178035608 | 178036817 | mouse | downregulated | heart | 0.0001 | GSE58455 | predicted associations | |
| lncRNA | 2 | 180399163 | 180401012 | mouse | downregulated | heart | 0.00005 | GSE58455 | predicted associations | |
| lncRNA | 3 | 52844748 | 53065601 | mouse | upregulated | heart | 0.00015 | GSE52313 | predicted associations | |
| lncRNA | 3 | 53734881 | 53735435 | mouse | downregulated | heart | 0.0025 | GSE58455 | predicted associations | |
| lncRNA | 3 | 55046501 | 55281788 | mouse | upregulated | heart | 0.00005 | GSE52313 | predicted associations | |
| lncRNA | 3 | 79688284 | 79750424 | mouse | upregulated | heart | 0.0002 | GSE52313 | predicted associations | |
| lncRNA | 3 | 83570249 | 83578234 | mouse | upregulated | heart | 0.00005 | GSE52313 | predicted associations | |
| lncRNA | 3 | 87885982 | 87912426 | mouse | upregulated | heart | 0.0006 | GSE52313 | predicted associations | |
| lncRNA | 3 | 87912760 | 87924210 | mouse | upregulated | heart | 0.00095 | GSE52313 | predicted associations | |
| lncRNA | 3 | 90416856 | 90457029 | mouse | upregulated | heart | 0.00045 | GSE52313 | predicted associations | |
| lncRNA | 3 | 95303120 | 95313291 | mouse | upregulated | heart | 0.00005 | GSE52313 | predicted associations | |
| lncRNA | 3 | 95330719 | 95360327 | mouse | upregulated | heart | 0.00005 | GSE52313 | predicted associations | |
| lncRNA | 3 | 115514229 | 115514620 | mouse | downregulated | heart | 0.0011 | GSE58455 | predicted associations | |
| lncRNA | 3 | 115704485 | 115704889 | mouse | upregulated | heart | 0.0049 | GSE58455 | predicted associations | |
| lncRNA | 3 | 129505348 | 129505616 | mouse | downregulated | heart | 0.00035 | GSE58455 | predicted associations | |
| lncRNA | 3 | 141902555 | 141949526 | mouse | upregulated | heart | 0.00005 | GSE52313 | predicted associations | |
| lncRNA | 3 | 145421713 | 145557241 | mouse | upregulated | heart | 0.00005 | GSE52313 | predicted associations | |
| lncRNA | 4 | 3192394 | 3192694 | mouse | upregulated | heart | 0.0027 | GSE58455 | predicted associations | |
| lncRNA | 4 | 12133869 | 12134101 | mouse | upregulated | heart | 0.00005 | GSE58455 | predicted associations | |
| lncRNA | 4 | 12135850 | 12136498 | mouse | upregulated | heart | 0.00005 | GSE58455 | predicted associations | |
| lncRNA | 4 | 32792877 | 32793233 | mouse | upregulated | heart | 0.0007 | GSE58455 | predicted associations | |
| lncRNA | 4 | 34550573 | 34550864 | mouse | upregulated | heart | 0.00035 | GSE58455 | predicted associations | |
| lncRNA | 4 | 40980116 | 40995293 | mouse | downregulated | heart | 0.00045 | GSE52313 | predicted associations | |
| lncRNA | 4 | 49548919 | 49562357 | mouse | downregulated | heart | 0.00005 | GSE52313 | predicted associations | |
| lncRNA | 4 | 58448147 | 58566823 | mouse | upregulated | heart | 0.0004 | GSE52313 | predicted associations | |
| lncRNA | 4 | 62936979 | 62996016 | mouse | upregulated | heart | 0.00045 | GSE52313 | predicted associations | |
| lncRNA | 4 | 63075951 | 63156194 | mouse | downregulated | heart | 0.00015 | GSE52313 | predicted associations | |
| lncRNA | 4 | 117791141 | 117791550 | mouse | downregulated | heart | 0.001 | GSE58455 | predicted associations | |
| lncRNA | 4 | 117880831 | 117963857 | mouse | upregulated | heart | 0.0003 | GSE52313 | predicted associations | |
| lncRNA | 4 | 129190892 | 129193208 | mouse | upregulated | heart | 0.00035 | GSE52313 | predicted associations | |
| lncRNA | 4 | 129724650 | 129776521 | mouse | upregulated | heart | 0.00035 | GSE52313 | predicted associations | |
| lncRNA | 4 | 134736250 | 134736917 | mouse | downregulated | heart | 0.0028 | GSE58455 | predicted associations | |
| lncRNA | 4 | 141414047 | 141431322 | mouse | upregulated | heart | 0.00005 | GSE52313 | predicted associations | |
| lncRNA | 4 | 149434755 | 149435057 | mouse | upregulated | heart | 0.00025 | GSE58455 | predicted associations | |
| lncRNA | 4 | 150229186 | 150243160 | mouse | upregulated | heart | 0.00005 | GSE52313 | predicted associations | |
| lncRNA | 5 | 4753888 | 4758131 | mouse | upregulated | heart | 0.00005 | GSE52313 | predicted associations | |
| lncRNA | 5 | 20878499 | 20884257 | mouse | upregulated | heart | 0.00005 | GSE52313 | predicted associations | |
| lncRNA | 5 | 25265787 | 25356505 | mouse | downregulated | heart | 0.00055 | GSE52313 | predicted associations | |
| lncRNA | 5 | 31215784 | 31223757 | mouse | upregulated | heart | 0.00005 | GSE52313 | predicted associations | |
| lncRNA | 5 | 32438117 | 32460207 | mouse | upregulated | heart | 0.00005 | GSE52313 | predicted associations | |
| lncRNA | 5 | 104034768 | 104058413 | mouse | upregulated | heart | 0.00035 | GSE52313 | predicted associations | |
| lncRNA | 5 | 109906117 | 109906338 | mouse | upregulated | heart | 0.0002 | GSE58455 | predicted associations | |
| lncRNA | 5 | 110703230 | 110715173 | mouse | downregulated | heart | 0.00005 | GSE52313 | predicted associations | |
| lncRNA | 5 | 111490892 | 111492382 | mouse | downregulated | heart | 0.0034 | GSE58455 | predicted associations | |
| lncRNA | 5 | 122046401 | 122068948 | mouse | downregulated | heart | 0.0009 | GSE52313 | predicted associations | |
| lncRNA | 5 | 122452986 | 122453215 | mouse | downregulated | heart | 0.00355 | GSE58455 | predicted associations | |
| lncRNA | 5 | 122990411 | 123064574 | mouse | downregulated | heart | 0.00005 | GSE52313 | predicted associations | |
| lncRNA | 5 | 135178464 | 135223252 | mouse | upregulated | heart | 0.00005 | GSE52313 | predicted associations | |
| lncRNA | 5 | 135791107 | 135791307 | mouse | downregulated | heart | 0.00195 | GSE58455 | predicted associations | |
| lncRNA | 5 | 137443648 | 137451686 | mouse | upregulated | heart | 0.0009 | GSE52313 | predicted associations | |
| lncRNA | 5 | 138046333 | 138056071 | mouse | upregulated | heart | 0.00045 | GSE52313 | predicted associations | |
| lncRNA | 6 | 9889904 | 9890155 | mouse | downregulated | heart | 0.00255 | GSE58455 | predicted associations | |
| lncRNA | 6 | 31067754 | 31068183 | mouse | downregulated | heart | 0.00195 | GSE58455 | predicted associations | |
| lncRNA | 6 | 31070575 | 31070864 | mouse | downregulated | heart | 0.00425 | GSE58455 | predicted associations | |
| lncRNA | 6 | 31074446 | 31074770 | mouse | downregulated | heart | 0.00325 | GSE58455 | predicted associations | |
| lncRNA | 6 | 31075486 | 31076005 | mouse | downregulated | heart | 0.00005 | GSE58455 | predicted associations | |
| lncRNA | 6 | 31078291 | 31078893 | mouse | downregulated | heart | 0.00075 | GSE58455 | predicted associations | |
| lncRNA | 6 | 31079564 | 31079993 | mouse | downregulated | heart | 0.00045 | GSE58455 | predicted associations | |
| lncRNA | 6 | 31088565 | 31089000 | mouse | downregulated | heart | 0.00045 | GSE58455 | predicted associations | |
| lncRNA | 6 | 31089416 | 31091935 | mouse | downregulated | heart | 0.00005 | GSE58455 | predicted associations | |
| lncRNA | 6 | 31092445 | 31093156 | mouse | downregulated | heart | 0.00005 | GSE58455 | predicted associations | |
| lncRNA | 6 | 31219975 | 31220312 | mouse | downregulated | heart | 0.004 | GSE58455 | predicted associations | |
| lncRNA | 6 | 31340676 | 31341424 | mouse | downregulated | heart | 0.00315 | GSE58455 | predicted associations | |
| lncRNA | 6 | 37277275 | 37392153 | mouse | upregulated | heart | 0.00005 | GSE52313 | predicted associations | |
| lncRNA | 6 | 42300030 | 42308461 | mouse | upregulated | heart | 0.0007 | GSE52313 | predicted associations | |
| lncRNA | 6 | 48986572 | 49006775 | mouse | upregulated | heart | 0.00005 | GSE52313 | predicted associations | |
| lncRNA | 6 | 55120619 | 55125014 | mouse | downregulated | heart | 0.00005 | GSE52313 | predicted associations | |
| lncRNA | 6 | 109505118 | 109505479 | mouse | downregulated | heart | 0.00005 | GSE58455 | predicted associations | |
| lncRNA | 6 | 113722095 | 113722602 | mouse | downregulated | heart | 0.00415 | GSE58455 | predicted associations | |
| lncRNA | 6 | 122463252 | 122480474 | mouse | upregulated | heart | 0.00005 | GSE52313 | predicted associations | |
| lncRNA | 6 | 124791118 | 124807346 | mouse | upregulated | heart | 0.00005 | GSE52313 | predicted associations | |
| lncRNA | 6 | 134931748 | 134936914 | mouse | upregulated | heart | 0.00005 | GSE52313 | predicted associations | |
| lncRNA | 6 | 136820957 | 136824490 | mouse | upregulated | heart | 0.00005 | GSE52313 | predicted associations | |
| lncRNA | 7 | 4877379 | 4896409 | mouse | upregulated | heart | 0.00005 | GSE52313 | predicted associations | |
| lncRNA | 7 | 25155167 | 25170136 | mouse | upregulated | heart | 0.00085 | GSE52313 | predicted associations | |
| lncRNA | 7 | 25247110 | 25261049 | mouse | upregulated | heart | 0.0001 | GSE52313 | predicted associations | |
| lncRNA | 7 | 30755632 | 30813266 | mouse | upregulated | heart | 0.0007 | GSE52313 | predicted associations | |
| lncRNA | 7 | 48124277 | 48124551 | mouse | downregulated | heart | 0.00005 | GSE58455 | predicted associations | |
| lncRNA | 7 | 65913676 | 66084935 | mouse | upregulated | heart | 0.00015 | GSE52313 | predicted associations | |
| lncRNA | 7 | 106301825 | 106331230 | mouse | downregulated | heart | 0.00035 | GSE52313 | predicted associations | |
| lncRNA | 7 | 141074898 | 141156475 | mouse | upregulated | heart | 0.00005 | GSE52313 | predicted associations | |
| lncRNA | 8 | 10984718 | 11008513 | mouse | upregulated | heart | 0.00005 | GSE52313 | predicted associations | |
| lncRNA | 8 | 11198420 | 11449288 | mouse | upregulated | heart | 0.00095 | GSE52313 | predicted associations | |
| lncRNA | 8 | 19704475 | 19705206 | mouse | downregulated | heart | 0.0017 | GSE58455 | predicted associations | |
| lncRNA | 8 | 24522066 | 24560139 | mouse | upregulated | heart | 0.00005 | GSE52313 | predicted associations | |
| lncRNA | 8 | 35580117 | 35580520 | mouse | downregulated | heart | 0.0022 | GSE58455 | predicted associations | |
| lncRNA | 8 | 35580875 | 35581229 | mouse | downregulated | heart | 0.00045 | GSE58455 | predicted associations | |
| lncRNA | 8 | 46970834 | 47005203 | mouse | upregulated | heart | 0.0005 | GSE52313 | predicted associations | |
| lncRNA | 8 | 72478005 | 72479946 | mouse | downregulated | heart | 0.0002 | GSE58455 | predicted associations | |
| lncRNA | 8 | 73017083 | 73027952 | mouse | upregulated | heart | 0.00025 | GSE52313 | predicted associations | |
| lncRNA | 8 | 79424108 | 79768941 | mouse | downregulated | heart | 0.00005 | GSE52313 | predicted associations | |
| lncRNA | 8 | 84205065 | 84206489 | mouse | downregulated | heart | 0.00005 | GSE58455 | predicted associations | |
| lncRNA | 8 | 84206753 | 84207447 | mouse | downregulated | heart | 0.0014 | GSE58455 | predicted associations | |
| lncRNA | 8 | 84855427 | 84927111 | mouse | downregulated | heart | 0.00095 | GSE52313 | predicted associations | |
| lncRNA | 8 | 90063483 | 90063709 | mouse | upregulated | heart | 0.00005 | GSE58455 | predicted associations | |
| lncRNA | 8 | 95350255 | 95377319 | mouse | upregulated | heart | 0.00005 | GSE52313 | predicted associations | |
| lncRNA | 8 | 95689683 | 95795863 | mouse | downregulated | heart | 0.00005 | GSE52313 | predicted associations | |
| lncRNA | 8 | 122333116 | 122364435 | mouse | upregulated | heart | 0.00095 | GSE52313 | predicted associations | |
| lncRNA | 9 | 5345453 | 5372614 | mouse | upregulated | heart | 0.0003 | GSE52313 | predicted associations | |
| lncRNA | 9 | 7347414 | 7358597 | mouse | upregulated | heart | 0.00005 | GSE52313 | predicted associations | |
| lncRNA | 9 | 20574389 | 20619498 | mouse | upregulated | heart | 0.00005 | GSE52313 | predicted associations | |
| lncRNA | 9 | 20820414 | 20833383 | mouse | upregulated | heart | 0.00035 | GSE52313 | predicted associations | |
| lncRNA | 9 | 45178257 | 45204239 | mouse | upregulated | heart | 0.00005 | GSE52313 | predicted associations | |
| lncRNA | 9 | 58135540 | 58161111 | mouse | upregulated | heart | 0.00005 | GSE52313 | predicted associations | |
| lncRNA | 9 | 62525755 | 62631761 | mouse | upregulated | heart | 0.00005 | GSE52313 | predicted associations | |
| lncRNA | 9 | 79446812 | 79566350 | mouse | upregulated | heart | 0.00005 | GSE52313 | predicted associations | |
| lncRNA | 9 | 83818764 | 84017847 | mouse | downregulated | heart | 0.00015 | GSE52313 | predicted associations | |
| lncRNA | 9 | 90051206 | 90056568 | mouse | downregulated | heart | 0.00005 | GSE52313 | predicted associations | |
| lncRNA | 9 | 90160255 | 90160631 | mouse | downregulated | heart | 0.0047 | GSE58455 | predicted associations | |
| lncRNA | 9 | 90161321 | 90161664 | mouse | downregulated | heart | 0.0015 | GSE58455 | predicted associations | |
| lncRNA | 9 | 101341568 | 101348017 | mouse | downregulated | heart | 0.00005 | GSE58455 | predicted associations | |
| lncRNA | 9 | 102910337 | 102991576 | mouse | upregulated | heart | 0.0004 | GSE52313 | predicted associations | |
| lncRNA | 9 | 105590170 | 105730323 | mouse | downregulated | heart | 0.00015 | GSE52313 | predicted associations | |
| lncRNA | 9 | 107583836 | 107612658 | mouse | downregulated | heart | 0.00005 | GSE52313 | predicted associations | |
| lncRNA | 9 | 114698496 | 114753409 | mouse | downregulated | heart | 0.00085 | GSE52313 | predicted associations | |
| lncRNA | 9 | 119517238 | 119533294 | mouse | downregulated | heart | 0.0002 | GSE52313 | predicted associations | |
| lncRNA | 9 | 122084996 | 122203526 | mouse | downregulated | heart | 0.00005 | GSE52313 | predicted associations | |
| lncRNA | 9 | 123698629 | 123761117 | mouse | downregulated | heart | 0.00005 | GSE52313 | predicted associations | |
| lncRNA | 10 | 40284277 | 40284645 | mouse | downregulated | heart | 0.0025 | GSE58455 | predicted associations | |
| lncRNA | 10 | 42654740 | 42680549 | mouse | upregulated | heart | 0.0007 | GSE52313 | predicted associations | |
| lncRNA | 10 | 56967788 | 56968148 | mouse | downregulated | heart | 0.0008 | GSE58455 | predicted associations | |
| lncRNA | 10 | 65005081 | 65006013 | mouse | downregulated | heart | 0.00005 | GSE58455 | predicted associations | |
| lncRNA | 10 | 65006893 | 65008492 | mouse | downregulated | heart | 0.00005 | GSE58455 | predicted associations | |
| lncRNA | 10 | 65008924 | 65010162 | mouse | downregulated | heart | 0.00005 | GSE58455 | predicted associations | |
| lncRNA | 10 | 76514944 | 76629294 | mouse | upregulated | heart | 0.00005 | GSE52313 | predicted associations | |
| lncRNA | 10 | 78157209 | 78158981 | mouse | downregulated | heart | 0.00125 | GSE58455 | predicted associations | |
| lncRNA | 10 | 79240002 | 79245495 | mouse | upregulated | heart | 0.00015 | GSE52313 | predicted associations | |
| lncRNA | 10 | 79519588 | 79565455 | mouse | upregulated | heart | 0.00025 | GSE52313 | predicted associations | |
| lncRNA | 10 | 80295583 | 80306705 | mouse | downregulated | heart | 0.0007 | GSE52313 | predicted associations | |
| lncRNA | 10 | 95325866 | 95326089 | mouse | downregulated | heart | 0.00205 | GSE58455 | predicted associations | |
| lncRNA | 10 | 97028028 | 97035338 | mouse | upregulated | heart | 0.00005 | GSE52313 | predicted associations | |
| lncRNA | 10 | 118000677 | 118001168 | mouse | downregulated | heart | 0.0009 | GSE58455 | predicted associations | |
| lncRNA | 10 | 126975092 | 127058037 | mouse | upregulated | heart | 0.00035 | GSE52313 | predicted associations | |
| lncRNA | 11 | 5761874 | 5772093 | mouse | upregulated | heart | 0.00005 | GSE52313 | predicted associations | |
| lncRNA | 11 | 16871220 | 16908717 | mouse | upregulated | heart | 0.00025 | GSE52313 | predicted associations | |
| lncRNA | 11 | 28753216 | 28826719 | mouse | downregulated | heart | 0.00005 | GSE52313 | predicted associations | |
| lncRNA | 11 | 50415430 | 50621161 | mouse | upregulated | heart | 0.00005 | GSE52313 | predicted associations | |
| lncRNA | 11 | 55206852 | 55233743 | mouse | upregulated | heart | 0.00005 | GSE52313 | predicted associations | |
| lncRNA | 11 | 65643369 | 65643764 | mouse | downregulated | heart | 0.0012 | GSE58455 | predicted associations | |
| lncRNA | 11 | 69759022 | 69759250 | mouse | upregulated | heart | 0.00005 | GSE58455 | predicted associations | |
| lncRNA | 11 | 70055590 | 70069358 | mouse | downregulated | heart | 0.0009 | GSE52313 | predicted associations | |
| lncRNA | 11 | 90197819 | 90252116 | mouse | downregulated | heart | 0.00005 | GSE52313 | predicted associations | |
| lncRNA | 11 | 104469362 | 104532110 | mouse | upregulated | heart | 0.00005 | GSE52313 | predicted associations | |
| lncRNA | 11 | 105829268 | 105851268 | mouse | upregulated | heart | 0.00005 | GSE52313 | predicted associations | |
| lncRNA | 11 | 117827450 | 117830507 | mouse | upregulated | heart | 0.00085 | GSE52313 | predicted associations | |
| lncRNA | 12 | 4587220 | 4587603 | mouse | downregulated | heart | 0.00005 | GSE58455 | predicted associations | |
| lncRNA | 12 | 48554188 | 48554553 | mouse | downregulated | heart | 0.00005 | GSE58455 | predicted associations | |
| lncRNA | 12 | 81268200 | 81361318 | mouse | upregulated | heart | 0.00005 | GSE52313 | predicted associations | |
| lncRNA | 12 | 86124161 | 86216845 | mouse | upregulated | heart | 0.00005 | GSE52313 | predicted associations | |
| lncRNA | 12 | 102988145 | 103057412 | mouse | upregulated | heart | 0.00005 | GSE52313 | predicted associations | |
| lncRNA | 12 | 105576731 | 105652538 | mouse | upregulated | heart | 0.00005 | GSE52313 | predicted associations | |
| lncRNA | 12 | 109544619 | 109569138 | mouse | upregulated | heart | 0.0008 | GSE52313 | predicted associations | |
| lncRNA | 12 | 110876076 | 110899807 | mouse | upregulated | heart | 0.00005 | GSE52313 | predicted associations | |
| lncRNA | 13 | 24800140 | 24801653 | mouse | upregulated | heart | 0.00095 | GSE58455 | predicted associations | |
| lncRNA | 13 | 32792703 | 32869673 | mouse | downregulated | heart | 0.00005 | GSE52313 | predicted associations | |
| lncRNA | 13 | 32973289 | 33031105 | mouse | upregulated | heart | 0.00045 | GSE52313 | predicted associations | |
| lncRNA | 13 | 41405347 | 41582665 | mouse | upregulated | heart | 0.0006 | GSE52313 | predicted associations | |
| lncRNA | 13 | 49639817 | 49662933 | mouse | upregulated | heart | 0.00005 | GSE52313 | predicted associations | |
| lncRNA | 13 | 73955905 | 73985051 | mouse | upregulated | heart | 0.00005 | GSE52313 | predicted associations | |
| lncRNA | 13 | 94827774 | 94965343 | mouse | upregulated | heart | 0.00005 | GSE52313 | predicted associations | |
| lncRNA | 13 | 96370960 | 96388544 | mouse | upregulated | heart | 0.0006 | GSE52313 | predicted associations | |
| lncRNA | 13 | 98011138 | 98022969 | mouse | upregulated | heart | 0.00005 | GSE52313 | predicted associations | |
| lncRNA | 13 | 98990478 | 98990685 | mouse | downregulated | heart | 0.0002 | GSE58455 | predicted associations | |
| lncRNA | 13 | 100191428 | 100286488 | mouse | upregulated | heart | 0.00085 | GSE52313 | predicted associations | |
| lncRNA | 13 | 100699384 | 100699740 | mouse | downregulated | heart | 0.0001 | GSE58455 | predicted associations | |
| lncRNA | 13 | 102359481 | 102359836 | mouse | downregulated | heart | 0.00055 | GSE58455 | predicted associations | |
| lncRNA | 14 | 45374894 | 45375267 | mouse | downregulated | heart | 0.00425 | GSE58455 | predicted associations | |
| lncRNA | 14 | 50888924 | 50889536 | mouse | downregulated | heart | 0.00005 | GSE58455 | predicted associations | |
| lncRNA | 14 | 50890280 | 50890575 | mouse | downregulated | heart | 0.00125 | GSE58455 | predicted associations | |
| lncRNA | 14 | 70008982 | 70095632 | mouse | upregulated | heart | 0.00005 | GSE52313 | predicted associations | |
| lncRNA | 14 | 70874214 | 70920042 | mouse | upregulated | heart | 0.0002 | GSE52313 | predicted associations | |
| lncRNA | 14 | 78560308 | 78560871 | mouse | downregulated | heart | 0.0043 | GSE58455 | predicted associations | |
| lncRNA | 14 | 124059339 | 124375994 | mouse | upregulated | heart | 0.00005 | GSE52313 | predicted associations | |
| lncRNA | 15 | 8584178 | 8660386 | mouse | upregulated | heart | 0.0002 | GSE52313 | predicted associations | |
| lncRNA | 15 | 31622281 | 31622588 | mouse | downregulated | heart | 0.0019 | GSE58455 | predicted associations | |
| lncRNA | 15 | 44437917 | 44438255 | mouse | downregulated | heart | 0.00135 | GSE58455 | predicted associations | |
| lncRNA | 15 | 55139173 | 55352354 | mouse | upregulated | heart | 0.00005 | GSE52313 | predicted associations | |
| lncRNA | 15 | 70064036 | 70064272 | mouse | upregulated | heart | 0.00035 | GSE58455 | predicted associations | |
| lncRNA | 15 | 78530971 | 78532888 | mouse | upregulated | heart | 0.00025 | GSE58455 | predicted associations | |
| lncRNA | 15 | 79346872 | 79358158 | mouse | upregulated | heart | 0.00005 | GSE52313 | predicted associations | |
| lncRNA | 15 | 82366111 | 82366331 | mouse | upregulated | heart | 0.00005 | GSE58455 | predicted associations | |
| lncRNA | 15 | 98985964 | 98989281 | mouse | upregulated | heart | 0.00005 | GSE58455 | predicted associations | |
| lncRNA | 15 | 99729059 | 99729513 | mouse | downregulated | heart | 0.0005 | GSE58455 | predicted associations | |
| lncRNA | 15 | 102966240 | 102971092 | mouse | downregulated | heart | 0.0003 | GSE52313 | predicted associations | |
| lncRNA | 16 | 10959410 | 10993263 | mouse | upregulated | heart | 0.00005 | GSE52313 | predicted associations | |
| lncRNA | 16 | 15887389 | 15888737 | mouse | upregulated | heart | 0.00005 | GSE52313 | predicted associations | |
| lncRNA | 16 | 18621849 | 18629982 | mouse | upregulated | heart | 0.00005 | GSE52313 | predicted associations | |
| lncRNA | 16 | 20128114 | 20129206 | mouse | downregulated | heart | 0.00125 | GSE58455 | predicted associations | |
| lncRNA | 16 | 20629874 | 20646495 | mouse | upregulated | heart | 0.00005 | GSE52313 | predicted associations | |
| lncRNA | 16 | 23468518 | 23535150 | mouse | upregulated | heart | 0.00035 | GSE52313 | predicted associations | |
| lncRNA | 16 | 30259851 | 30283311 | mouse | downregulated | heart | 0.0001 | GSE52313 | predicted associations | |
| lncRNA | 16 | 32271546 | 32274837 | mouse | downregulated | heart | 0.00085 | GSE52313 | predicted associations | |
| lncRNA | 16 | 32410390 | 32410932 | mouse | downregulated | heart | 0.00005 | GSE58455 | predicted associations | |
| lncRNA | 16 | 37777107 | 37836611 | mouse | upregulated | heart | 0.00005 | GSE52313 | predicted associations | |
| lncRNA | 16 | 45093500 | 45130419 | mouse | upregulated | heart | 0.00005 | GSE52313 | predicted associations | |
| lncRNA | 16 | 56477958 | 56690357 | mouse | upregulated | heart | 0.00025 | GSE52313 | predicted associations | |
| lncRNA | 16 | 56806404 | 56886114 | mouse | upregulated | heart | 0.00005 | GSE52313 | predicted associations | |
| lncRNA | 16 | 57624375 | 57754845 | mouse | upregulated | heart | 0.00005 | GSE52313 | predicted associations | |
| lncRNA | 16 | 85794068 | 85803345 | mouse | upregulated | heart | 0.00005 | GSE52313 | predicted associations | |
| lncRNA | 16 | 90921841 | 90922140 | mouse | upregulated | heart | 0.0004 | GSE58455 | predicted associations | |
| lncRNA | 16 | 92346179 | 92359613 | mouse | upregulated | heart | 0.00035 | GSE52313 | predicted associations | |
| lncRNA | 16 | 92392197 | 92466365 | mouse | upregulated | heart | 0.00005 | GSE52313 | predicted associations | |
| lncRNA | 17 | 7929430 | 8020128 | mouse | upregulated | heart | 0.00005 | GSE52313 | predicted associations | |
| lncRNA | 17 | 12878385 | 12878766 | mouse | downregulated | heart | 0.00015 | GSE58455 | predicted associations | |
| lncRNA | 17 | 12879699 | 12880460 | mouse | downregulated | heart | 0.00025 | GSE58455 | predicted associations | |
| lncRNA | 17 | 13018136 | 13020192 | mouse | downregulated | heart | 0.00005 | GSE58455 | predicted associations | |
| lncRNA | 17 | 13060080 | 13074161 | mouse | downregulated | heart | 0.00085 | GSE52313 | predicted associations | |
| lncRNA | 17 | 23697249 | 23697947 | mouse | upregulated | heart | 0.00185 | GSE58455 | predicted associations | |
| lncRNA | 17 | 29230722 | 29237663 | mouse | upregulated | heart | 0.00005 | GSE52313 | predicted associations | |
| lncRNA | 17 | 31907876 | 31909054 | mouse | upregulated | heart | 0.00045 | GSE58455 | predicted associations | |
| lncRNA | 17 | 33911849 | 33918474 | mouse | upregulated | heart | 0.00005 | GSE52313 | predicted associations | |
| lncRNA | 17 | 35479346 | 35479599 | mouse | upregulated | heart | 0.00005 | GSE58455 | predicted associations | |
| lncRNA | 17 | 45563368 | 45563634 | mouse | downregulated | heart | 0.0014 | GSE58455 | predicted associations | |
| lncRNA | 17 | 78851978 | 78852255 | mouse | upregulated | heart | 0.00005 | GSE58455 | predicted associations | |
| lncRNA | 17 | 80933181 | 80933449 | mouse | downregulated | heart | 0.00455 | GSE58455 | predicted associations | |
| lncRNA | 17 | 86163622 | 86164767 | mouse | downregulated | heart | 0.0005 | GSE58455 | predicted associations | |
| lncRNA | 17 | 86165533 | 86165786 | mouse | downregulated | heart | 0.0049 | GSE58455 | predicted associations | |
| lncRNA | 18 | 9312591 | 9313319 | mouse | downregulated | heart | 0.0034 | GSE58455 | predicted associations | |
| lncRNA | 18 | 11018025 | 11019001 | mouse | downregulated | heart | 0.00115 | GSE58455 | predicted associations | |
| lncRNA | 18 | 12957174 | 12957474 | mouse | downregulated | heart | 0.0013 | GSE58455 | predicted associations | |
| lncRNA | 18 | 61488982 | 61489216 | mouse | upregulated | heart | 0.00155 | GSE58455 | predicted associations | |
| lncRNA | 18 | 89839635 | 89839850 | mouse | upregulated | heart | 0.00005 | GSE58455 | predicted associations | |
| lncRNA | 19 | 4184383 | 4189316 | mouse | downregulated | heart | 0.00005 | GSE52313 | predicted associations | |
| lncRNA | 19 | 5298180 | 5308727 | mouse | downregulated | heart | 0.0002 | GSE52313 | predicted associations | |
| lncRNA | 19 | 5741830 | 5773901 | mouse | upregulated | heart | 0.0009 | GSE52313 | predicted associations | |
| lncRNA | 19 | 11395837 | 11410613 | mouse | upregulated | heart | 0.00005 | GSE52313 | predicted associations | |
| lncRNA | 19 | 11593027 | 11605692 | mouse | upregulated | heart | 0.00025 | GSE52313 | predicted associations | |
| lncRNA | 19 | 11661047 | 11679301 | mouse | upregulated | heart | 0.00075 | GSE52313 | predicted associations | |
| lncRNA | 19 | 23166967 | 23167460 | mouse | downregulated | heart | 0.00005 | GSE58455 | predicted associations | |
| lncRNA | 19 | 24043885 | 24044133 | mouse | downregulated | heart | 0.0012 | GSE58455 | predicted associations | |
| lncRNA | 19 | 27321268 | 27321734 | mouse | downregulated | heart | 0.0012 | GSE58455 | predicted associations | |
| lncRNA | 19 | 29088727 | 29089097 | mouse | downregulated | heart | 0.00235 | GSE58455 | predicted associations | |
| lncRNA | 19 | 29999621 | 30035145 | mouse | upregulated | heart | 0.00005 | GSE52313 | predicted associations | |
| lncRNA | 19 | 34809941 | 34810556 | mouse | downregulated | heart | 0.00095 | GSE58455 | predicted associations | |
| lncRNA | 19 | 37342979 | 37418505 | mouse | upregulated | heart | 0.0002 | GSE52313 | predicted associations | |
| lncRNA | 19 | 44407800 | 44421925 | mouse | upregulated | heart | 0.00005 | GSE52313 | predicted associations | |
| lncRNA | 19 | 56739285 | 56740042 | mouse | downregulated | heart | 0.0007 | GSE58455 | predicted associations | |
| lncRNA | 19 | 56750795 | 56751206 | mouse | downregulated | heart | 0.00385 | GSE58455 | predicted associations | |
| lncRNA | 19 | 56752060 | 56752762 | mouse | downregulated | heart | 0.00035 | GSE58455 | predicted associations | |
| lncRNA | 19 | 56753713 | 56754510 | mouse | downregulated | heart | 0.0008 | GSE58455 | predicted associations | |
| lncRNA | 19 | 57403398 | 57404213 | mouse | upregulated | heart | 0.00055 | GSE58455 | predicted associations | |
| lncRNA | 19 | 61304840 | 61305257 | mouse | downregulated | heart | 0.00265 | GSE58455 | predicted associations | |
| lncRNA | X | 24008412 | 24008758 | mouse | downregulated | heart | 0.00005 | GSE58455 | predicted associations | |
| lncRNA | X | 33842433 | 33842723 | mouse | upregulated | heart | 0.0001 | GSE58455 | predicted associations | |
| lncRNA | X | 157817884 | 157818189 | mouse | upregulated | heart | 0.00345 | GSE58455 | predicted associations | |
| lncRNA | X | 168653671 | 168654030 | mouse | downregulated | heart | 0.0017 | GSE58455 | predicted associations | |
| lncRNA | 1 | 33327716 | 33337996 | human | downregulated | heart | 0.0003 | GSE71613 | predicted associations | |
| lncRNA | 1 | 151336663 | 151345184 | human | downregulated | heart | 0.00005 | GSE71613 | predicted associations | |
| lncRNA | 1 | 203274602 | 203278903 | human | downregulated | heart | 0.00005 | GSE71613 | predicted associations | |
| lncRNA | 2 | 69083494 | 69098740 | human | upregulated | heart | 0.00025 | GSE71613 | predicted associations | |
| lncRNA | 2 | 113885079 | 113891703 | human | downregulated | heart | 0.0001 | GSE71613 | predicted associations | |
| lncRNA | 2 | 232786602 | 232791096 | human | downregulated | heart | 0.00025 | GSE71613 | predicted associations | |
| lncRNA | 3 | 108028954 | 108029292 | human | downregulated | heart | 0.00055 | GSE71613 | predicted associations | |
| lncRNA | 3 | 184741019 | 184741422 | human | upregulated | heart | 0.0004 | GSE71613 | predicted associations | |
| lncRNA | 4 | 6695619 | 6698948 | human | downregulated | heart | 0.0001 | GSE71613 | predicted associations | |
| lncRNA | 4 | 52471898 | 52472175 | human | upregulated | heart | 0.001 | GSE71613 | predicted associations | |
| lncRNA | 4 | 56329029 | 56329403 | human | upregulated | heart | 0.00005 | GSE71613 | predicted associations | |
| lncRNA | 4 | 143228505 | 143228802 | human | upregulated | heart | 0.00135 | GSE71613 | predicted associations | |
| lncRNA | 5 | 5699148 | 5699452 | human | upregulated | heart | 0.00075 | GSE71613 | predicted associations | |
| lncRNA | 5 | 71490373 | 71492744 | human | downregulated | heart | 0.0001 | GSE71613 | predicted associations | |
| lncRNA | 5 | 71493495 | 71505514 | human | downregulated | heart | 0.00015 | GSE71613 | predicted associations | |
| lncRNA | 5 | 95220803 | 95297596 | human | downregulated | heart | 0.00015 | GSE71613 | predicted associations | |
| lncRNA | 5 | 172741771 | 172755551 | human | downregulated | heart | 0.00005 | GSE71613 | predicted associations | |
| lncRNA | 7 | 5566144 | 5571380 | human | downregulated | heart | 0.0004 | GSE71613 | predicted associations | |
| lncRNA | 7 | 123674785 | 123675027 | human | upregulated | heart | 0.0013 | GSE71613 | predicted associations | |
| lncRNA | 8 | 120372452 | 120373483 | human | upregulated | heart | 0.00005 | GSE71613 | predicted associations | |
| lncRNA | 9 | 34709006 | 34710208 | human | downregulated | heart | 0.0001 | GSE71613 | predicted associations | |
| lncRNA | 9 | 40601488 | 40601745 | human | downregulated | heart | 0.0002 | GSE71613 | predicted associations | |
| lncRNA | 11 | 330371 | 330642 | human | downregulated | heart | 0.001 | GSE71613 | predicted associations | |
| lncRNA | 11 | 18287810 | 18291521 | human | downregulated | heart | 0.00005 | GSE71613 | predicted associations | |
| lncRNA | 11 | 66081955 | 66084645 | human | downregulated | heart | 0.00005 | GSE71613 | predicted associations | |
| lncRNA | 11 | 107571933 | 107590573 | human | downregulated | heart | 0.00005 | GSE71613 | predicted associations | |
| lncRNA | 12 | 110583824 | 110584750 | human | downregulated | heart | 0.00135 | GSE71613 | predicted associations | |
| lncRNA | 13 | 90273846 | 90276331 | human | downregulated | heart | 0.00005 | GSE71613 | predicted associations | |
| lncRNA | 14 | 75744038 | 75749866 | human | downregulated | heart | 0.00015 | GSE71613 | predicted associations | |
| lncRNA | 14 | 95078722 | 95093337 | human | downregulated | heart | 0.00005 | GSE71613 | predicted associations | |
| lncRNA | 15 | 80253248 | 80263562 | human | downregulated | heart | 0.00005 | GSE71613 | predicted associations | |
| lncRNA | 16 | 56623286 | 56624997 | human | downregulated | heart | 0.00005 | GSE71613 | predicted associations | |
| lncRNA | 16 | 56651282 | 56652732 | human | downregulated | heart | 0.00005 | GSE71613 | predicted associations | |
| lncRNA | 17 | 80186357 | 80198073 | human | downregulated | heart | 0.00005 | GSE71613 | predicted associations | |
| lncRNA | 19 | 2476131 | 2478332 | human | downregulated | heart | 0.00005 | GSE71613 | predicted associations | |
| lncRNA | 19 | 7741908 | 7744798 | human | downregulated | heart | 0.00005 | GSE71613 | predicted associations | |
| lncRNA | 19 | 12902303 | 12904467 | human | downregulated | heart | 0.00005 | GSE71613 | predicted associations | |
| lncRNA | 19 | 18893583 | 18902152 | human | upregulated | heart | 0.00005 | GSE71613 | predicted associations | |
| lncRNA | 19 | 39818997 | 39832177 | human | downregulated | heart | 0.0002 | GSE71613 | predicted associations | |
| lncRNA | 22 | 30658825 | 30662890 | human | downregulated | heart | 0.0003 | GSE71613 | predicted associations | |
| lncRNA | 22 | 39055423 | 39055665 | human | upregulated | heart | 0.00135 | GSE71613 | predicted associations | |
| lncRNA | X | 77070541 | 77071054 | human | upregulated | heart | 0.00005 | GSE71613 | predicted associations | |
